# Measurement and documentation of quality indicators for the end-of-life care of hospital patients a nationwide retrospective record review study

**DOI:** 10.1186/s12904-023-01299-x

**Published:** 2023-11-08

**Authors:** F. M. Bijnsdorp, B. Schouten, A. K. L. Reyners, C. Wagner, A. L. Francke, S. M. van Schoten

**Affiliations:** 1 Nivel, Netherlands Institute For Health Services Research, Utrecht, the Netherlands; 2 Department Of Public And Occupational Health, Amsterdam Umc, Vrije Universiteit Amsterdam, Amsterdam Public Health Research Institute, P/O Box 7057, Amsterdam, MB 1007 the Netherlands; 3 Department of Medical Oncology, Center of Expertise in Palliative Care, University Medical Centre Groningen, University of Groningen, Groningen, the Netherlands

**Keywords:** Palliative care, End of life care, Healthcare quality, Healthcare quality indicators, Hospitals

## Abstract

**Background:**

Quality of care at the end of life in hospitals is often perceived to be lower compared to the care that is provided to people who die in their own home. Documenting and measuring indicators of common end-of-life symptoms could help improve end-of-life care in hospitals. This study provided insight into quality indicators for the end-of-life care of patients who died in a Dutch hospital, and assessed differences between deceased patients who were admitted for palliative/terminal care versus patients admitted for other reasons.

**Methods:**

In a retrospective record review study, trained nurses reviewed electronic health records (EHRs) of patients who died in 2019 (*n* = 2998), in a stratified sample of 20 Dutch hospitals. The nurses registered whether data was found in de EHRs about quality indicators for end-of-life care. This concerned: symptoms (pain, shortness of breath, anxiety, depressive symptoms), spiritual and psychological support and advance care planning. Multilevel regression analyses were performed to assess differences between patients who had been admitted for palliative/terminal care and patients admitted for other reasons.

**Results:**

Common end-of-life symptoms were rarely measured using a standardized method (e.g. Numeric Rating Scale, Visual Analogue Scale or Utrecht Symptom Diary). The symptom burden of pain was measured using a standardized method more often (63.3%) than the symptom burden of shortness of breath (2.2%), anxiety (0.5%) and depressive symptoms (0.3%). Similarly, little information was documented in the EHRs regarding wish to involve a spiritual counsellor, psychologist or social worker. Life expectancy was documented in 66%. The preferred place of death was documented less often (20%). The documentation of some quality indicators differed between patients who were admitted for palliative/terminal care compared to other patients.

**Conclusion:**

Except for the burden of pain, symptoms are rarely measured with standardized methods in patients who died in Dutch Hospitals. This study underlines the importance of documenting information about symptom burden and aspects related to advance care planning, and spiritual and psychological support to improve the quality of end-of-life care for patients in hospitals. Furthermore, uniformity in measuring methods improves the possibility to compare results between patient groups and settings.

**Supplementary Information:**

The online version contains supplementary material available at 10.1186/s12904-023-01299-x.

## Background

Although most people at the end of life wish to remain at home until death [1], about 37–54% of deaths in Western countries occur in a hospital [2]. In the Netherlands, this percentage is about 20% [3–5]. The quality of care at the end of life, is perceived as less good compared to the care that is provided to people who die in their own home, as experienced by their relatives [6]. Increasing attention has been paid in national and international policy, practice and research to improving the quality of care for patients who stay in hospital and die there. Good quality end-of-life care requires the prevention and alleviation of suffering, and the identification and careful assessment and treatment of symptoms and problems in the physical, psychological, social and spiritual dimensions [7, 8].

The chance that a patient receives good end-of-life care is greater when healthcare professionals realize in good time that the patient is nearing death. Having timely advance care planning discussions with the patient about their care needs and preferences at the end of life also improves end-of-life care [9, 10]. However, a nationwide study by Melin-Johansson et al. has shown that about a third of patients at the end of life did not have advance care planning discussions with a physician. Not having these conversations was associated with dying in a hospital instead of dying at home [11]. It can be assumed that people whose death is expected, because they suffer and will eventually die from an illness relevant to palliative care, will have more advance care planning discussions and a higher quality of end-of-life care than people who die unexpectedly.

Given that the quality of care at the end of life is rated lower in hospitals compared to other care settings such as hospices and the patient’s own home [6], it is important to gain more insight into the quality of care at the end of life in hospitals. This could be done by looking at quality indicators that are relevant for end-of-life care and assessing whether and how these quality indicators are documented in the electronic health records (EHRs) of patients who died in a hospital [12]. Quality indicators are defined as measurable elements of care that provide an indication of the quality of care [12, 13]. Which quality indicators provide a good indication of the quality of care at the end of life has been widely studied [12, 14–18]. A Dutch 2009 study provided a core set of quality indicators in palliative care in the Netherlands [18]. Moreover, since 2017 the Netherlands has adopted the Quality Framework for Palliative Care [19]. This framework was developed by Dutch expert stakeholders, and includes uniform quality requirement for end-of-life care regarding 10 dimensions. Among these 10 dimensions, and in the Dutch core set of quality indicators for palliative care, are the quality indicators of interest in our study. These include the identification and management of common end-of-life symptoms such as pain, shortness of breath, anxiety and depressive symptoms. In addition, aspects related to psychological or spiritual support (e.g. the involvement of a spiritual counsellor when a patient has expressed a wish for this) and advance care planning are also indicators of good quality end-of-life care, as included in the Quality Framework for Palliative Care and international studies [12, 14–19].

In order to avoid imposing extra documentation work and a research burden among professionals, patients and relatives, it is recommended to reuse routine health data from EHRs for research on quality indicators. In a previous pilot study, Lokker and colleagues have shown that some quality indicators of end-of-life care could be assessed using the EHRs of cancer patients who died in Dutch hospitals [16]. However, so far, quality indicators for end-of-life care have not been extracted on a large scale from the EHRs of a wider group of patients who died in the hospital (i.e. broader than deceased cancer patients).

Quality indicators are not only relevant for patients who were admitted for palliative/terminal care, but also for patients who were (initially) admitted for curative treatments or reasons other than palliative/terminal care and/or whose death was relatively unexpected. We hypothesized that, in general, the EHRs of deceased patients who had been admitted for palliative/terminal care would contain more documented information about aspects of advance care planning, symptom measurements and symptom relief than the EHRs of other deceased patients. However, even in a palliative care population, delivering timely and good end-of-life care could be difficult, especially in hospital settings predominantly focusing on curative treatments, life-saving and resuscitation [20]. This could result into care needs remaining overlooked at the end of life [20, 21]. Hence, it is important to evaluate the quality of end-of-life care of patients who die in the hospital and to compare quality indicators between patients admitted for palliative/terminal care and patients admitted for other reasons. In addition, more insight is needed in whether and how the nearing end of life was identified and documented in the EHRs. This knowledge could help hospital professionals, particularly medical and nursing staff, improve the quality of end-of-life care. The following research questions were formulated:

(i) What are the outcomes for quality indicators for end-of-life care among patients who died in a hospital in the Netherlands?
(ii) To what extent do these outcomes differ between deceased hospital patients who had been admitted for palliative/terminal care and deceased hospital patients who had been admitted for other reasons?

## Methods

### Study design and sample

The data collection in the current study was part of the Dutch Monitor on Adverse Events in hospitals, a longitudinal retrospective record review study among patients who died in Dutch hospitals [22]. For the Dutch Monitor on Adverse Events study, a stratified sample was drawn from 20 of the 74 hospitals in the Netherlands, comprising university hospitals (*n* = 4), tertiary teaching hospitals (*n* = 6) and general hospitals (*n* = 10). Relatively many university hospitals were included in the sample to allow comparisons between the three types of hospitals. To correct for this oversampling of university hospitals, the overall results were weighted by hospital type. Per hospital, about 150 records of patients who had died in the hospital in 2019 were randomly selected from the hospital information system. EHRs from patients admitted to the psychiatry or obstetrics department and EHRs of children younger than one year were excluded.

In total, 2998 EHRs were included of patients who died in hospital in 2019. Comparison between characteristics of the sample and the characteristics for all patients who died in Dutch hospitals showed that the sample in the current study was representative for the main characteristics (i.e. age and sex of the patient, admission length, medical specialism and hospital type and region) for all admissions. Detailed information on the design of the study and weighting procedure was published previously [22].

### Record review

 The EHRs were assessed and reviewed by registered nurses (*n* = 17) and physicians (*n* = 8; medical specialties: surgery, internal medicine and neurology), who were trained in the systematic method of record review which is based on the Harvard Medical Practice Study [23]. The full procedure of the record review in the Dutch Monitor on Adverse Events is described in more detail elsewhere [24]. For the purpose of the present study, some questions on end-of-life care were added to the assessment form for the nurse record review. The nurses used an assessment form developed by the researchers to assess the extent to which data regarding quality indicators were measured and subsequently documented in the EHRs. Table 1 includes a frame with the quality indicators of interest and the corresponding dimensions of the Dutch Quality Framework for Palliative Care.

The reviewers did not assess patient EHRs from hospitals where they worked or had worked in the past. The reviewing of the patient EHRs by the nurses took place on location in the participating hospital or, if possible and permitted by the hospital, remotely by logging into the hospital’s digital system. During the assessment of data in the patient EHRs, the researchers were available for specific questions about the assessment procedures or for further explanation of the questions regarding indicators for end-of-life care. In order to increase inter-rater validity, meetings with the reviewing nurses were organized by the research team on a regular basis to discuss questions or difficulties regarding the assessment form with respect to e.g. symptom measurements and outcomes.

**Table 1:** Quality indicators frame

Dimension in quality framework	Theme in quality framework	Quality indicators in this study
Structure and process	Aspects of advance care planning	• % patients for whom information is documented on aspects relating to advance care planning and impending death: 1. Live expectancy was documented; 2. Preferred place of death was documented; 3. Preference on resuscitation, ventilation and treatment (restrictions) were documented
Physical, psychological, social and spiritual dimensions	Symptom burden	• % patients for whom a standardised pain measurement was documented and also the outcome (symptom burden); • % patients for whom a standardised shortness of breath measurement was documented and also the outcome (symptom burden); • % patients for whom a standardised anxiety measurement was documented and also the outcome (symptom burden); • % patients for whom a standardised depressive symptoms/sombre mood measurement was documented and also the outcome (symptom burden); • % patients for whom was documented whether a spiritual counsellor was wished for and involved. • % patients for whom was documented whether a psychologist or social worker was wished for and involved.

### Inter-rater reliability

The reliability of this study was assessed using 297 patient records (10% of the total sample). Each record was reviewed by two nurses. The percentage of agreement between the two rating nurses varied between 67.9%, for the documentation of the life expectancy or prognosis, and 99.7%, for the documentation of depressive symptoms/sombre mood [25]. The reliability of the current study was moderate to good.

### Measurements

#### Admitted for palliative/terminal care versus other admission reasons

In the Netherlands, palliative care is an integral part of regular hospital care, and therefore not a distinct medical specialty [26]. Thus subgroups were defined based on admission reason. In the assessment forms, the nurses had to register the reason for the hospital admission. They used data in the EHRs to determine whether a patient: (i) had been admitted for palliative care (i.e. admitted for palliative care or palliative treatment: yes/no) and/or terminal care (i.e. in terminal stage on admission: yes/no), or (ii) had been admitted (initially) for a curative treatment and/or other reasons for admission than palliative/terminal care.

#### Quality indicators regarding symptoms

The nurses also reviewed the EHRs with respect to aspects related to the identification and management of a number of common end-of-life symptoms, including pain, shortness of breath, anxiety and depressive symptoms/sombre mood [12, 15, 19]. The nurses registered whether or not pain, shortness of breath, anxiety and depressive symptoms/sombre mood were measured with a standardized method at least once in the seven days prior to the patient’s death. If the hospital stay was shorter than seven days, the data for the total number of days following admission were registered. Standardized methods included the Numeric Pain Rating Scale (NRS) [27], the Visual Analogue Scale (VAS) [28] and the Utrecht Symptom Diary (USD), which is a Dutch adaptation of the Edmonton Symptom Assessment Scale (ESAS) [29]. These measurements were all considered standardized methods, as they are internationally widely known and used and as these are most commonly used in Dutch practice [18, 19]. The NRS, VAS and USD could be answered using an 11-point scale, ranging from 0 = no symptom burden to 10 = very high symptom burden. If the symptom burden was documented with another standardized method, nurses had to note the name of this method in the assessment form. The researchers provided a manual including a step-by-step guide for assessing symptom burden and an overview of the methods that were considered standardized and non-standardized (see Supplementary file 1). If the symptom burden was measured with a standardized method, nurses noted in the assessment form: (i) on how many days of the seven days prior to the patient’s death the symptom burden was measured and (ii) on how many days the symptom burden was ≥ 4 according to the NRS, VAS or USD. A cut-off point of 4 is commonly used to identify people with clinically relevant levels of symptom burden who require an intervention [18]. If symptom burden was not measured using a standardized method, the nurses noted whether the symptom burden had been considered in some other way, and if so, how this was documented.

#### Quality indicators for psychological and spiritual support

The nurses also recorded whether or not information about the patient’s wish to involve a spiritual counsellor in the fourteen days prior to the patient’s death was documented in the patient’s EHRs (yes/no/unknown). If this was documented and the patient wanted a spiritual counsellor, nurses recorded whether a spiritual counsellor was actually involved (yes/no/unknown). Comparable questions were asked about the desired and actual involvement of a psychologist and a social worker.

#### Quality indicators associated with advance care planning

The nurses recorded whether or not several aspects relevant for advance care planning were documented in the patient EHRs. The aspects of advance care planning were, as the other quality indicators, based on the Dutch Quality Framework Palliative Care. These included: the patient’s life expectancy or prognosis, the preferred place of death, wishes regarding whether or not to resuscitate, and wishes regarding forgoing, starting, stopping or continuing medical treatments (yes/no).

#### Background characteristics

Relevant background characteristics of the patient and the admission included: sex of the patient (male/female), patient age (in years), admission status (elective, acute, transfer, other), admission department, admission diagnoses, not to be resuscitated (NTBR) policy upon admission, number of days in the intensive care (IC) unit and whether the patient received palliative sedation (yes/no and starting date).

### Data analysis

Data from the assessment forms were first analysed using descriptive statistics. With regard to symptom burden, the percentages of patients with a documented standardized score for pain, shortness of breath, anxiety and depressive symptoms/sombre mood were analysed, as well as the associated scores for the symptom burden.

In addition, the percentage of patients with documented wishes for the involvement of a spiritual counsellor, psychologist and social worker were calculated. If such a wish was documented, the percentages of cases in which these professionals were actually involved were also calculated. Next, the percentages of patients with documented aspects of advance care planning were calculated.

Furthermore, differences between patients who had been admitted for palliative/terminal care and patients with other admission reasons were analysed and compared for each quality indicator. Multilevel regression analyses were performed taking account of EHRs (level 1) nested within hospital departments (level 2) and hospital organizations (level 3). The multilevel analyses were adjusted for the hospital type and the length of the hospital admission (fixed effects). Prior to the multilevel analyses, symptom burden was dichotomized into yes/no, whereby yes = measured at least once with a standardized method (NRS/VAS/USD) and no = all other categories. STATA version 14 was used for all analyses.

### Ethics

The office of the Medical Ethical Review Committee of Amsterdam University Medical Centers (IRB00002991) reviewed the study protocol and declared that this study was not subject to the Medical Research Involving Human Subjects Act (reference no.2020.052). Therefore, no further formal medical ethical approval was required [30]. Moreover, this study was conducted under the Dutch Healthcare Quality, Complaints and Disputes Act, with quality improvement as the primary aim. Therefore, the study was exempt from the requirement of individual informed consent.

## Results

The sample characteristics are presented in Table 2. About 22% of the deceased patients whose EHRs were assessed were admitted for palliative/terminal care. The median age of these patients was 78 years and about 45% were female. The median duration of the hospital admission until death was shorter for patients who were admitted for palliative/terminal care (2 days) compared to those who were admitted for other reasons (5 days). Acute admissions occurred more frequently among patients who were admitted for palliative/terminal care (95.4%) compared to those who were admitted for other reasons (86.8%). More patients who were admitted for palliative/terminal care received palliative sedation (43%) compared to other patients (38%).

**Table 2:** Descriptive characteristics of patients who were admitted for palliative/terminal care compared to patients who were admitted for other reasons and who died in hospital

	**Total ** **(***n* **= 2998)**	**Admitted for palliative/terminal care** **(***n* **= 647)**	**Admitted for other reasons** **(***n* **= 2351)**
**Age, median [IQR]**	78 [69–85]	78 [67–86]	78 [69–85]
**Female (%)**	45.3	47.3	44.7
**Duration of hospital admission (in days), median [IQR]**	4 [2–10]	2 [1–5]	5 [2–11]
**Acute admission (%)**	88.6	95.4	86.8
**Palliative sedation (%)**	39.1	43.0	38.0

## Quality indicators for symptom burden

### Documentation of pain measurement and pain burden

In the seven days prior to the patient’s death, attention had been paid to the symptom burden of pain in almost three quarters of the patients who were admitted for palliative/terminal care (see Table 3). This is less than in patients who were admitted for other reasons (83%). Multilevel analysis showed that the odds of pain being measured at least once with the NRS, VAS or USD in the seven days prior to the patient’s death were significantly lower for patients who were admitted for palliative/terminal care compared to patients who were admitted for other reasons (OR 0.63, 95% CI [0.50, 0.78]).

About 18% of the EHRs of patients who were admitted for palliative/terminal care show evidence of attention paid to the symptom burden of pain in a different, non-standardized way, compared to 11.2% among the other group of patients. Healthcare professionals had paid attention to pain in various ways, i.e. observing the patient, discussing pain with the patient, nurse or medical doctor, or pain medication was documented in the patient EHRs. In addition, the nurse reviewers recorded that it was sometimes impossible to measure pain with a standardized method because of the state of the patient (e.g. in a coma or sedated).

On average, pain was measured in 86.4% of days during the hospital stay among the subgroup in which pain was measured with a standardized method (*n* = 1898). The score was equal to or higher than 4 on about 24.3% of these days (see Table 3). Multilevel analysis showed that the percentage of days during the hospital stay on which pain was measured with the NRS, VAS or USD did not significantly differ between the patient groups (B -0.03, 95% CI [-0.06, 0.00]). On average, patients who were admitted for palliative/terminal care had a significant higher percentage of days in which they had a score of 4 or more out of the total number of days on which pain was measured (30.7%) than other patients (23%) (B 0.07, 95% CI [0.01, 0.12]).

**Table 3:** Quality indicators for measuring the symptom burden of pain

	**Total ** **(*n*** **= 2998)**	**Admitted for palliative/terminal care ** **(*n*** ** = 647)**	**Admitted for other reasons** **(*n*** **= 2351)**	**OR/B (95% CI)**
Yes, symptom burden was measured at least once with a standardized method (NRS/VAS/USD) (%)	63.3	49.7	66.8	OR 0.63 (0.50–0.78)
No, but symptom burden was measured at least once with another standardized method (%)	4.9	5.3	4.8	
No, but attention was paid to the symptom burden in a different way (%)	12.6	18.1	11.2	
No, symptom burden was not measured (%)	19.2	26.9	17.3	
% of days measured out of the total hospital stay, M (SD)	86.4 (24.4)	84.5 (25.0)	86.8 (24.2)	B -0.03 (-0.06-0.00)^a^
% of days of a score ≥ 4 out of the days measured, M (SD)	24.3 (36.3)	30.7 (41.1)	23 (35.1)	B 0.07 (0.01–0.12)^a^

^a^Because the distribution of the % of days was somewhat skewed, a sensitivity analysis (including a logistic multilevel regression analysis) was performed. The results of the sensitivity analysis pointed in the same direction as the reported linear multilevel analysis

### Measurement and documentation of symptom burden of shortness of breath

Only about 2% of all the patients had recorded measurements of the symptom burden of shortness of breath with the NRS, VAS or USD at least once in the seven days prior to the patient’s death (see Table 4). In about a quarter of the patients who were admitted for palliative/terminal care, the symptom burden was measured with another standardized method, which was smaller compared to the patients who were admitted for other reasons (37%). Among the patients who were admitted for palliative/terminal care, attention had been paid to shortness of breath in a different way somewhat more often (46%) compared to other patients (41.3%). Analysis of the open text fields showed that healthcare professionals paid attention to shortness of breath in various ways, including observation, discussing shortness of breath with patients, nurses and medical doctors, monitoring saturation, or documenting medication for shortness of breath. The nurses who assessed the patient records indicated that it was sometimes impossible to measure shortness of breath with a standardized method because of the patient’s condition (e.g.in a coma or sedated). In 27.3% of the EHRs of patients who were admitted for palliative/terminal care, the symptom burden of shortness of breath had not been measured at all, compared to 19.3% of the other patients. Due to the small number of patients where this symptom burden was measured at least once with a standardized method, it was not possible to perform multilevel analysis to compare groups.

On average, shortness of breath was measured with the NRS, VAS or USD on 80.8% of the days during the total hospital admission among the subgroup in which this was measured with these standardized methods (*n* = 66); the score was equal to or higher than 4 on about 34.8% of these days (see Table 4). If the score is above 4, follow-up treatment is necessary to relieve the symptoms.

**Table 4:** Quality indicators for measuring the symptom burden of shortness of breath

	**Total ** **(*n*** ** = 2998)**	**Admitted for palliative/terminal care ** **(*n*** ** = 647)**	**Admitted for other reasons** **(*n*** ** = 2351)**
Yes, symptom burden was measured at least once with a standardized method (NRS/VAS/USD) (%)	2.2	1.4	2.4
No, but symptom burden was measured at least once with another standardized method (%)	34.5	24.7	37.0
No, but attention was paid to the symptom burden in a different way (%)	42.3	46.3	41.3
No, symptom burden has not been measured (%)	21.0	27.6	19.3
% of days measured out of the total hospital admission, M (SD)	80.8 (29.4)	^a^	^a^
% of days with a score ≥ 4 out of the days measured, M (SD)	34.8 (42.8)	^a^	^a^

^a^Number of patients too small to calculate mean and SD

### Documentation of anxiety measurement and symptom burden

Less than 1% of the patient EHRs had recorded measurements of anxiety with a standardized method (NRS, VAS, USD) at least once in the last seven days prior to the patient’s death (see Table 5). More than half of the patients had recorded measurements of anxiety in some other way. In about one in three of the EHRs of patients who were admitted for palliative/terminal care, there was evidence of attention paid to anxiety in a different way. This was less compared to the other group of patients (37.6%). Anxiety was considered in the open text fields of patient EHRs in the form of documentation of conversations between healthcare professionals and patients, notes that the patient expressed being afraid of something, observations of the patient and documentation of medication for anxiety. On average, anxiety was measured with the NRS, VAS or USD on 65.9% of the days during the hospital admission among the subgroup in which anxiety was measured with these standardized methods (*n* = 15); the score was equal to or higher than 4 on about 27.8% of these days (see Table 5).

**Table 5:** Quality indicators for measuring the symptom burden of anxiety

	Total (*n* = 2998)	Admitted for palliative/terminal care (*n* = 647)	Admitted for other reasons (*n* = 2351)
Yes, symptom burden was measured at least once with a standardized method (NRS/VAS/USD) (%)	0.5	0.3	0.6
No, but symptom burden was measured at least once with another standardized method (%)	2.3	3.2	2.0
No, but attention was paid to the symptom burden in a different way (%)	36.5	32.1	37.6
No, symptom burden has not been measured (%)	60.7	64.4	59.8
% of days measured out of the total hospital admission, M (SD)	65.9 (38.3)	^a^	^a^
% of days with a score ≥ 4 out of the days measured, M (SD)	27.8 (46.1)	^a^	^a^

^a^Number of patients too low to calculate mean and SD

### Measurement and documentation of burden of depressive symptoms/sombre mood

Almost none of the patient EHRs had records showing depressive symptoms/sombre mood had been measured at least once in the seven days prior to the patient’s death with a standardized method (NRS, VAS or USD) (see Table 6). Also, this symptom burden was measured at least once with another standardized method in about 2% of the patient EHRs. In 18.5% of the EHRs of patients who were admitted for palliative/terminal care, there was evidence of attention paid to depressive symptoms/sombre mood in a different way. This was a smaller proportion compared to the EHRs of patients who were admitted for other reasons (25.2%).

On average, depressive symptoms/sombre mood were measured with the NRS, VAS or USD on 57.9% of the hospital stay days among the subgroup in which this symptom burden was measured with these standardized methods (*n* = 9); the score was equal to or higher than 4 on about 21.2% of these days.

**Table 6:** Quality indicators for measuring the symptom burden of depressive symptoms/sombre mood

	**Total ** **(*n*** ** = 2998)**	**Admitted for palliative/terminal care** **(*n*** ** = 647)**	**Admitted for other reasons** **(*n*** ** = 2351)**
Yes, symptom burden was measured at least once with a standardized method (NRS/VAS/USD) (%)	0.3	0.1	0.3
No, but symptom burden was measured at least once with another standardized method (%)	2.1	2.9	1.9
No, but attention was paid to the symptom burden in a different way (%)	23.8	18.5	25.2
No, symptom burden has not been measured (%)	73.8	78.5	72.6
% of days measured out of the total hospital admission, M (SD)	57.9 (41.1)	^a^	^a^
% of days with a score ≥ 4 out of the days measured, M (SD)	21.2 (40.2)	^a^	^a^

^a^ Number of patients too low to calculate mean and SD

### Quality indicators for support of a spiritual counsellor, psychologist or social worker

In 12.5% of the EHRs of patients who were admitted for palliative/terminal care, it was documented that the patient wanted support of a spiritual counsellor (see Table 7). The actual involvement of a spiritual counsellor was documented in about 74% of these patients with an expressed wish. Among patients who were admitted for other reasons, the wish for support of a spiritual counsellor was documented in 15% of the EHRs. The involvement of a spiritual counsellor was documented in about 70% of these patients with an expressed wish. Multilevel analysis showed that the odds of a documented wish for a spiritual counsellor did not significantly differ between the patient groups (OR = 1.12, 95% CI [0.84, 1.50]). There was also no significant difference in the odds of documented information about the actual involvement of a spiritual counsellor between patients who were admitted for palliative/terminal care and patients who were admitted for other reasons (OR = 1.06, 95% CI [0.62, 1.83]).

A wish for guidance by a psychologist was documented in about 3% of the patients’ EHRs. In about 80% of these patients with an expressed wish, the involvement of a psychologist was documented. The wish for guidance by a social worker was documented in about 5% of the EHRs of patients who were admitted for palliative/terminal care. In almost 67% of these patients with an expressed wish, the involvement of a social worker was documented. Among patients who were admitted for other reasons, about 9% had a documented wish for guidance by a social worker. The involvement of a social worker was documented in about 68% of these patients’ EHRs. The number of patients with a documented wish for guidance by a psychologist or social worker was too low to perform multilevel analysis.

**Table 7:** Quality indicators for guidance by spiritual counsellor, psychologist or social worker

	Total (*n* = 2998)		Admitted for palliative/terminal care (*n* = 647)		Admitted for other reasons (*n* = 2351)		OR (95% CI)	
	**Wished**	**involved**	**Wished**	**involved**	**Wished**	**involved**	**wished**	**involved**
**Spiritual counsellor (%)**	14.4	71.2	12.5	73.9	15.0	70.6	1.12 (0.84–1.50)	1.45 (0.78–2.68)
**Psychologist (%)**	2.9	78.0	2.1	79.1	3.1	77.8	^a^	^a^
**Social worker (%)**	8.3	67.8	5.1	66.8	9.1	67.9	^a^	^a^

^a^Number of patients with documented wish too low to estimate OR

### Quality indicators for advance care planning and the impending death

The life expectancy was documented in about three quarter of the EHRs of patients who were admitted for palliative/terminal care (see Table 8). This was more often than in the EHRs of other patients (63.1%). In addition, the preferred place of death was documented in a greater proportion of patients who were admitted for palliative/terminal care (30.5%) compared to other patients (17.7%). Multilevel analyses showed that patients who were admitted for palliative/terminal care had significantly higher odds of having their life expectancy (OR 2.19, 95% CI [1.74, 2.76]) and preferred place of death (OR 3.24, 95% CI [2.58, 4.08]) documented in their patient EHRs. About 60% of the patients who were admitted for palliative/terminal care had a documented ‘do not resuscitate, do not ventilate’ policy in their patient EHRs, which was higher compared to other patients (41.4%).

**Table 8:** Identification of impending death and other aspects of advanced care planning

	Total (*n* = 2998)	Admitted for palliative/terminal care (*n* = 647)	Admitted for other reasons (*n* = 2351)	OR (95% CI)
Life expectancy documented (%)	65.7	75.8	63.1	2.19 (1.74–2.76)
Preferred place of death documented (%)	20.3	30.5	17.7	3.24 (2.58–4.08)
NTBR^a^ policy after 24 h of admission (%)				
No treatment restrictions	5.4	1.9	6.4	
Do not resuscitate	6.5	3.1	7.5	
Do not resuscitate, do not ventilate	23.2	19.8	24.1	
Abstinence from treatment	45.3	59.4	41.4	
Unknown	1.2	0.8	1.4	
Other	18.4	15.2	19.2	

^a^NTBR = ‘do not resuscitate, do not ventilate’ policy

## Discussion

### Main findings

Our study showed that some common end-of-life symptoms are recorded quite often, but are rarely measured using a standardized method (e.g. NRS, VAS, USD). However, the symptom burden of pain appeared to be measured more often using a standardized method (63.3%) than the symptom burden of shortness of breath (2.2%), anxiety (0.5%) and depressive symptoms/sombre mood (0.3%). This does not mean that patients don’t have these symptoms. Similarly, little information was documented in the EHRs on the wish to involve a spiritual counsellor, psychologist or social worker. Standardized measurements of pain were documented less often in the records of patients who were admitted for palliative/terminal care compared to other patients. A pain score of 4 or higher was more likely for patients who were admitted for palliative/terminal care compared to other patients.

That the EHRs of deceased patients relatively often included information about the symptom burden of pain, is in line with a previous study of older people receiving palliative care in Sweden that also found that pain was the most documented symptom and that anxiety and depressed mood were seldom documented [31]. In the Netherlands this exception might be explained by the general attention paid to pain measurement in hospital care since 2008, as part of a national patient safety programme [32].

Standardized measurement and documentation of particularly psychological or social problems is rare, even when patients were admitted for palliative/terminal care. This is remarkable given that palliative care aims to relieve suffering, not only physically, but also in the psychological, social and spiritual dimensions [8]. Moreover, standardized measurements of pain were documented less frequently in EHRs of patients who were admitted for palliative/terminal care compared to other patients [33].However, this does not necessarily mean that healthcare professionals did not think about identifying and relieving symptom burden among patients who were admitted for palliative/terminal care. The EHRs often included notes about pain, anxiety or depressive symptoms/sombre mood, showing that the healthcare professionals paid attention to the symptom burden in a different way. One explanation for the limited use of standardized methods to measure symptom burden among patients who were admitted for palliative/terminal care could be that these patients were unable to answer the questions in the validated instruments in the final days of life, for instance if the patient was unconscious due to a coma or palliative sedation. Other possible explanations require further investigation [34–36].

With regard to the scores on the quality indicators, a pain score of 4 or higher was documented more often among patients who were admitted for palliative/terminal care compared to other patients. This was contrary to our assumption that more attention would be paid to the measurement of pain, and subsequently more effective pain relief would be given, in patients admitted for palliative/terminal care. It could however be that pain relief was not possible for some of the patients who were admitted for palliative/terminal care. Nevertheless, proper symptom assessment and management might be challenging in settings predominantly focusing on curative treatment of the disease, life-saving and resuscitation [20]. Despite this primary focus on cure, healthcare professionals should also be able to provide good palliative care, including timely identification of pain or other symptoms, and prevention and alleviation of the symptom burden.

Very little information regarding the wish to involve a spiritual counsellor, psychologist or social worker was documented in the EHRs. For some patients this was not possible, for instance because the patient was unconscious, but for others this could be helpful and improve their care at the end of life. Moreover, when a wish for guidance by a spiritual counsellor, psychologist or social worker *was* documented, subsequent information about the actual involvement of these professionals was often lacking. It could be that the patient died shortly after expressing a wish for a spiritual counsellor, psychologist or social worker. It could also be that the professional was involved in practice but that this was not properly documented, or that this type of support was not readily available. It could thus be that there was a difference between the care that was delivered and the care that was documented.

Furthermore, the identification of the approaching death in patients who were initially not admitted for palliative/terminal care was documented in various ways, e.g. including discussion with the family about the patient’s approaching death, starting palliative sedation or using a care pathway for dying patients. As expected, the life expectancy or prognosis of the patient was documented more frequently in patients who were admitted for palliative/terminal care (75.8%) compared to patients who were admitted for other reasons (63.1%). Also the preferred place of death was more likely to be documented in patients who were admitted for palliative/terminal care compared with patients admitted for other reasons (30.5% versus 17.7%). However, it could be that in other cases the advance care planning discussions took place outside the hospital, for instance with the general practitioner or in nursing homes. This information is often not accessible in the EHR by healthcare professionals in the hospital. Therefore the percentage of patients who had advance care planning discussions might be underestimated. Although aspects related to advance care planning were documented for a substantial number of patients, the integration and documentation of aspects relevant for advance care planning (e.g. care needs and preferences) could be further improved by standardizing the terminology that medical and nursing staff use in this regard. To prevent an increase in the registration burden, it is important that aspects related to advance care planning, and also aspects related to other quality indicators discussed in this paper, are built into the software of the EHRs. Documenting in a standardized and uniform way could prevent a situation in which patients’ wishes are not honoured due to the absence of information or inconsistencies, and thus improve the quality of care [37].

### Methodological considerations

A strength of this nationwide retrospective record study is that we used a large and representative sample of patients who died in hospitals in the Netherlands. To our knowledge, this was the first study to provide indications of the quality of end-of-life care in hospitals on such a large scale. By using EHRs, we were able to include the clinical and administrative data of deceased patients who died in Dutch hospitals without imposing an additional registration burden beyond routine care.

A limitation is that the interpretation of the results depended on the documented information in the EHRs. For instance, it was not always possible to determine whether the patient was unresponsive, for example due to palliative sedation or other forms of reduced consciousness, which might have been a reason why the patient was not asked to rate the symptom burden.

Another limitation for the interpretation of the results concerns the fact that we used the term ‘quality indicators’, while the aspects examined were not fully elaborated quality indicators. Quality indicators are usually specified with a numerator, denominator and/or a performance standard [12]. However, these quality indicators, as well as most other quality indicators for end-of-life care, are currently lacking broadly accepted performance standards [4]. Because of this, we have to be cautious when interpreting the results, and no firm conclusions can be drawn on whether the indicator scores point to good, moderate or poor quality of end-of-life care.

### Implications for research, policy and practice

To improve the quality of end-of-life care in hospitals, it is recommended that symptom burden measurements are conducted using standardized methods and are then unambiguously documented in the patient EHRs. This will enable healthcare professionals to determine when additional treatment is needed and when to take additional measures. Moreover, this is important to provide a complete picture of the quality of end-of-life care for patients in hospitals, and subsequently to improve care where needed. Uniformity in registration in the EHRs would also facilitate the automated extraction of data on symptom burden at the end of life [38]. This would be an efficient and less time-consuming way to evaluate the quality of care at the end of life in hospitals.

It is also important to clarify which methods can be used in hospitals for people with a reduced level of consciousness who are no longer able to give a score, where nurses and doctors then have to rely on observations. There are validated observation scales (e.g. REPOS, PACSLAC-D and PAINAD [34–36]) to assess pain in people with cognitive impairment or a limited ability to communicate that may also be suitable for measuring pain in people with reduced consciousness at the end of life. These observation instruments are still barely used in Dutch hospitals. Future research could assess whether these instruments are valid and efficient for end-of-life care in the hospital setting.

As long as absolute, broadly accepted performance standards are lacking, it could be useful to set ‘best-practice performance standards’ in future research and policy to compare indicator scores of one hospital with national data and data from other hospitals. A best-practice performance standard could for instance be established for each quality indicator by identifying the upper quartile (the upper 25%) of hospitals with the best scores. The upper margin of the third quartile or above can then be defined as the best-practice performance standard [4, 39]. Setting such a relative best-practice performance standard could help pinpoint areas for improvement and encourage hospitals to work towards this relative standard [38].

Furthermore, the number of patients with an expressed and recorded wish for guidance from a spiritual counsellor, psychologist or social worker was relatively low among all the patients in the current study. It remains unclear whether this was because these patients did not want guidance or whether this was not discussed in the hospital. Information on whether this was discussed elsewhere was not available. It has been suggested that palliative care practice generally pays more attention to symptom management and less to the psychological and spiritual dimensions [12, 20]. It could thus be that it is not always clear to patients that these professionals are available to support them. It is therefore recommended that nurses discuss the possible role that spiritual counsellors, psychologists or social workers could have in supporting hospital patients at the end of life and their families. In addition, it could be helpful to document not only an expressed wish for the involvement of a spiritual counsellor, psychologist or social worker in the EHRs, but also the fact that the patient does not want this guidance if this is the case. Future research could further investigate why the involvement of spiritual counsellors, psychologists or social workers is relatively low among hospital patients at the end of life (e.g. is there no need for this guidance, do the healthcare professionals not discuss this option with patients, is there too little time to actually involve these professionals?).

## Conclusion

Except for the burden of pain, common end-of-life symptoms and aspects are rarely measured using a standardized method in patients who died in a hospital in the Netherlands. Pain is measured using a standardized method much more frequently compared to the symptoms of shortness of breath, anxiety and depressive symptoms/sombre mood. This study underlines the importance of uniformity in measuring, discussing and documenting symptom burden and aspects related to advance care planning and spiritual and psychological wellbeing in the EHRs in order to improve the quality of end-of-life care for patients who die in hospital.

### Supplementary Information

**Additional file 1.** Manual for nurse raters.

## Data Availability

Data are available upon reasonable request after approval of the corresponding author.

## Abbreviations

CI: Confidence interval
EHR: Electronic health records
ESAS: Edmonton Symptom Assessment Scale
IC: Intensive care
IQR: Inter quartile range
M: Mean
MC: Medium care
NRS: Numeric Pain Rating Scale
NTBR: Not to be resuscitated
OR: Odds ratio
PACSLAC-D: Pain Assessment Checklist for Seniors with Severe Dementia
PAINAID: Pain Assessment in Advanced Dementia Scale
REPOS: The Rotterdam Elderly Pain Observation Scale
SD: Standard deviation
USD: Utrecht Symptom Diary
VAS: the Visual Anologue Scale
