# Supplementary material for: Measurement and documentation of quality indicators for the end-of-life care of hospital patients a nationwide retrospective record review study
Source: BMC Palliat Care. 2023 Nov 8;22:174. doi: 10.1186/s12904-023-01299-x (PMC10631072; doi:10.1186/s12904-023-01299-x)
Supplement: Supplementary file 1 — Additional file 1. Manual for nurse raters. [file 12904_2023_1299_MOESM1_ESM.docx]

## Supplementary file 1 – Manual for nurse raters

Step-by-step schedule for filling in the symptom burden questions regarding the quality of end-of-life care:

1. Always select “Yes” if it was measured at least once in the past 7 days using NRS/VAS/USD.
2. Select “No, but it was measured at least once using another standardized method, namely...” if the method appears in the table of other standardized methods and was used in the past 7 days.
3. Select “No, but attention was paid to the symptom burden in some other way” if it was discussed specifically in the past 7 days. If this is not clear or if it depends on your interpretation, select the last option.
4. Select “No, no consideration at all was given to the symptom burden” if none of the above answers can be found in the file for the past 7 days.
5. The final column shows methods that are not seen as standardized methods for measuring the burden of that particular symptom.
6. If methods are used that are not yet included in the list, please send a message to [researcher]

**Important:** The Terminal Phase Clinical Pathway sometimes has scales of 0-10, which are seen as NRS. In that case, select “Yes, at least one measurement was performed in the past few days using NRS/VAS/USD”.

|  | Yes, at least one measurement was performed in the past few days using NRS/VAS/USD. | No, but at least one measurement was performed using another standardized method, namely... | No, but attention was paid to the symptom burden in some other way | No, no consideration at all was given to the symptom burden | Methods that are not considered standardized methods |
| --- | --- | --- | --- | --- | --- |
| Symptom burden |  |  |  |  |  |
| Pain | NRS/VAS/USD | PAINAD | The report contains some description of the symptom burden, for example: ‘no pain symptoms’ |  | Alert, Verbal, Pain, Unresponsive (AVPU) ‑ is used to measure consciousness, with pain stimuli applied to determine this. |
|  |  | Rotterdam Elderly Pain Observation Scale (REPOS) |  |  | Wakker, Aanspreekbaar, Pijngevoelig, A-reactief (WAPA) – same as AVPU but in Dutch. |
|  |  | Critical Care Pain Observation Tool (CPOT) |  |  |  |
|  |  | Terminal Phase Clinical Pathway* |  |  |  |
| * The Terminal Phase Clinical Pathway sometimes has scales of 0-10, which are seen as NRS. In that case, select “Yes, at least one measurement was performed in the past few days using NRS/VAS/USD.” | | | | | |
|  | **Yes, at least one measurement was performed in the past few days using NRS/VAS/USD.** | **No, but at least one measurement was performed using another standardized method, namely...** | **No, but attention was paid to the symptom burden in some other way** | **No, no consideration at all was given to the symptom burden** | **Methods that are not considered standardized methods** |
| Dyspnoea/shortness of breath | NRS/VAS/USD | Oxygen saturation (including telemetry for artificial lung respiration/ patients on a monitor) | The report contains some description of the symptom burden, for example: ‘patient is very short of breath’ |  | (Modified) Early Warning Score (MEWS/ EWS)  Vroeg Signalering Score (VSS) |
|  |  | Respiration rate per minute |  |  | Rotterdam Elderly Pain Observation Scale (REPOS) |
|  |  | Terminal Phase Clinical Pathway* |  |  | Stroke Impact Scale (SIS) |
|  |  |  |  |  | Alarm score (only reported in one hospital) |
|  |  |  |  |  | ABCDE method where A stands for *ademhaling* (‘respiration’ in Dutch) |
|  |  |  |  |  | Situation, Background, Assessment, Recommendation (SBAR/ SBARR) |
|  |  |  |  |  |  |

* The Terminal Phase Clinical Pathway sometimes has scales of 0-10, which are seen as NRS. In that case, select “Yes, at least one measurement was performed in the past few days using NRS/VAS/USD.”

|  | Yes, at least one measurement was performed in the past few days using NRS/VAS/USD. | No, but at least one measurement was performed using another standardized method, namely... | No, but attention was paid to the symptom burden in some other way | No, no consideration at all was given to the symptom burden | Methods that are not considered standardized methods |
| --- | --- | --- | --- | --- | --- |
| Anxiety |  | Terminal Phase Clinical Pathway* | The report contains some description of the symptom burden, for example: ‘the patient seems fearful’ |  | Delirium Observation Scale (DOS) |
|  |  |  |  |  | Rotterdam Elderly Pain Observation Scale (REPOS) |
|  |  |  |  |  | Identification of Elderly At Risk (ISAR) |
|  |  |  |  |  | Richmond Agitation-Sedation Scale (RASS) |
|  |  |  |  |  |  |
| Depressive symptoms/sombre mood |  | Terminal Phase Clinical Pathway* | The report contains some description of the symptom burden, for example: ‘the patient feels sombre about the situation’ |  | Delirium Observation Scale (DOS) |
|  |  |  |  |  | Identification of Elderly At Risk (ISAR) |

* The Terminal Phase Clinical Pathway sometimes has scales of 0-10, which are seen as NRS. In that case, select “Yes, at least one measurement was performed in the past few days using NRS/VAS/USD.”
